# Automated recording of home cage activity and temperature of individual rats housed in social groups: The Rodent Big Brother project

**DOI:** 10.1371/journal.pone.0181068

**Published:** 2017-09-06

**Authors:** William S. Redfern, Karen Tse, Claire Grant, Amy Keerie, David J. Simpson, John C. Pedersen, Victoria Rimmer, Lauren Leslie, Stephanie K. Klein, Natasha A. Karp, Rowland Sillito, Agis Chartsias, Tim Lukins, James Heward, Catherine Vickers, Kathryn Chapman, J. Douglas Armstrong

**Affiliations:** 1 Drug Safety and Metabolism, AstraZeneca R&D, Babraham Research Campus, Cambridge, United Kingdom; 2 Drug Safety and Metabolism, AstraZeneca R&D, Alderley Park, Cheshire, United Kingdom; 3 Quantitative Biology, IMED, AstraZeneca, Darwin Building (Unit 310), Cambridge Science Park, Cambridge, United Kingdom; 4 Actual Analytics Ltd, Edinburgh, United Kingdom; 5 NC3Rs, London, United Kingdom; 6 School of Informatics, University of Edinburgh, Appleton Tower, Edinburgh, United Kingdom; Radboud University Medical Centre, NETHERLANDS

## Abstract

Measuring the activity and temperature of rats is commonly required in biomedical research. Conventional approaches necessitate single housing, which affects their behavior and wellbeing. We have used a subcutaneous radiofrequency identification (RFID) transponder to measure ambulatory activity and temperature of individual rats when group-housed in conventional, rack-mounted home cages. The transponder location and temperature is detected by a matrix of antennae in a baseplate under the cage. An infrared high-definition camera acquires side-view video of the cage and also enables automated detection of vertical activity. Validation studies showed that baseplate-derived ambulatory activity correlated well with manual tracking and with side-view whole-cage video pixel movement. This technology enables individual behavioral and temperature data to be acquired continuously from group-housed rats in their familiar, home cage environment. We demonstrate its ability to reliably detect naturally occurring behavioral effects, extending beyond the capabilities of routine observational tests and conventional monitoring equipment. It has numerous potential applications including safety pharmacology, toxicology, circadian biology, disease models and drug discovery.

## Introduction

Behavioral studies in academic research and drug discovery are commonly conducted on rats, which are also the preferred rodent species for toxicology and safety pharmacology studies [1;2;3]. In drug discovery, such studies are used to inform critical decisions; in preclinical safety assessment they are required by regulatory authorities prior to human exposure [4]. Conventional approaches to recording the behavior of laboratory rats are limited to ‘snapshot’ assessments, whether for academic research or in the pharmaceutical industry. Typical examples include manual observations by a trained observer using multi-parameter assessments of global neurobehavioral effects (e.g., the Irwin test or Functional Observational Battery (FOB) [2;5;6;7;8;9;10], or a scoring scale for abnormal behaviors (e.g., stereotypies [11;12;13]; seizures [14]). For convenience, behavioral assessments are usually performed during the daytime, when these nocturnal animals are generally less active. Ambulatory activity measurements can be automated using photobeam arrays [15;16;17;18;19;20], a passive infrared sensor [21;22], a stabilimeter (‘rocking cage’/’jiggle box’) [15;23] or birds-eye videotracking [24;25;26;27]. A wider set of behaviors can be detected using pressure sensors beneath the cage [28;29;30] or by videoanalysis software [31]. These automated methods necessitate the rats, which are social animals [32;33], to be singly housed, typically in bespoke cages, and generally on bench tops rather than cage racks, requiring dedicated space. In mice, methods have been described using radiofrequency identity (RFID) transponders or differently colored fur dyes for recording ambulatory activity of individuals when group-housed [34;35;36;37;38;39]; with a recent exception [39], these required the use of bespoke cages. This presents a significantly greater challenge for rats, which are ~10 times larger in size, therefore with their RFID transponder potentially at a greater distance from the baseplate RFID reader (located beneath the cage floor) than with mice.

As well as general activity and behavior we are (or should be) interested in what is happening to the physiology of our rats: temperature, for example, is a key indicator of physiological homeostasis. Rats respond to ingestion of toxic agents by lowering their core temperature [40;41;42], which affects the absorption, metabolism and excretion of drugs [43] and their overall toxicity [40;41;42]. Larger decreases in temperature may reflect a more profound toxicity, a pharmacological effect on central thermoregulatory control, impaired metabolic heat production, vasodilatory heat loss, or a physiological response to hypoglycemia [44] or hypoxia [45]. Conversely, increases in temperature in rats may reveal other toxicological mechanisms, including skeletal muscle toxicity or an immunological response [46]. Core temperature can be recorded via a rectal thermistor or by radiotelemetry, both of which have their drawbacks. Measurement of rectal temperature requires manual restraint which itself elevates core temperature [47], whereas intraperitoneal radiotelemetry necessitates surgical laparotomy and is relatively expensive [42]. Other methods, e.g., a subcutaneous RFID transponder read-off through the cage wall via a hand-held transceiver [48;49;50] or infrared imaging [27], only enable manual, snapshot measurements.

**Fig 1:**
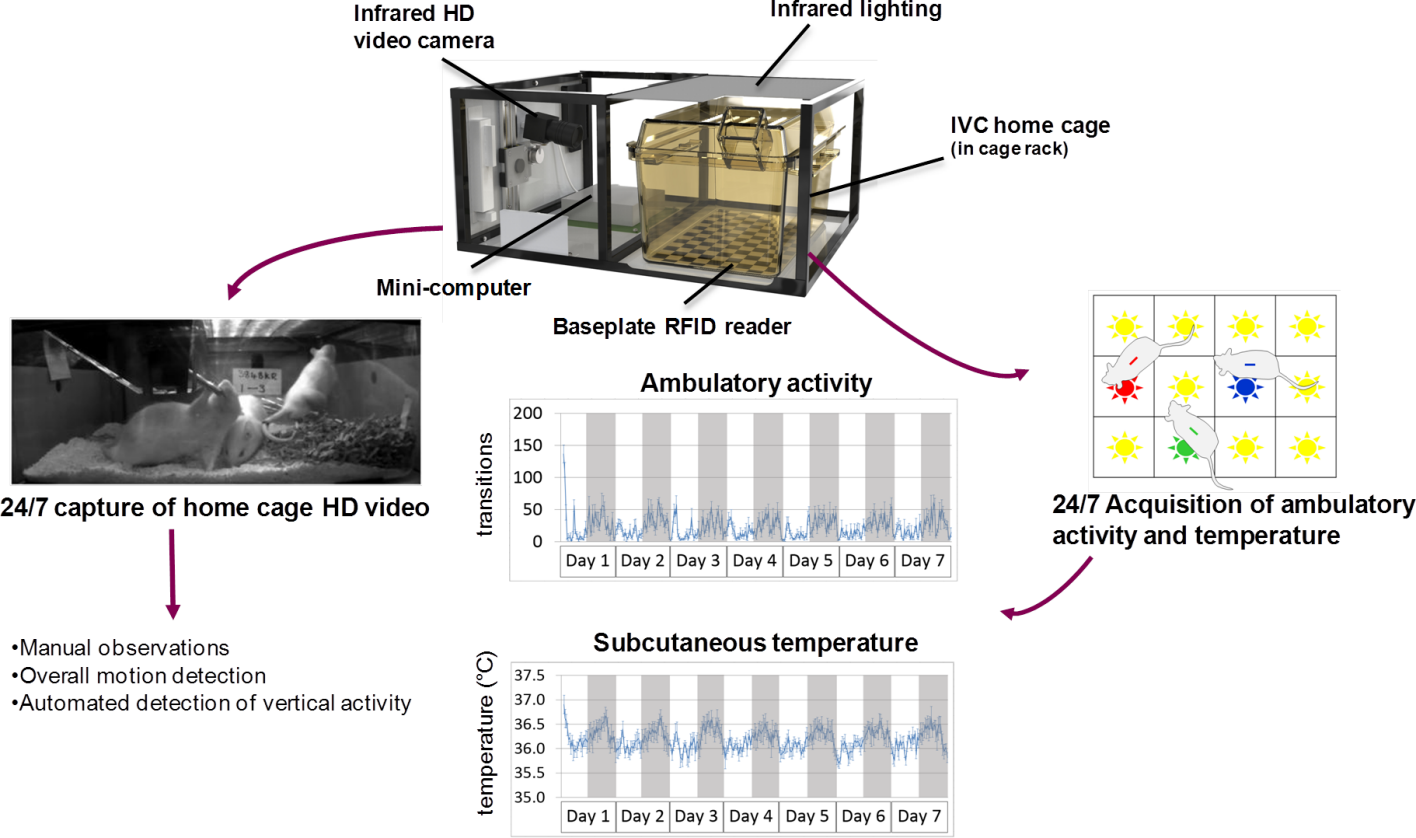
Schematic overview of the Rodent Big Brother (Home Cage Analyzer; ActualHCA™) system. Rats are housed in social groups in standard IVC cages with the Home Cage Analyzer equipment slotted inside an adjacent cage void. The sealed baseplate RFID reader derives positional and temperature information for each animal individually from their subcutaneous RFID chip. The infrared HD video camera captures 25 fps, infrared gray scale video, continuously. An array of infrared LEDs above the cage provides even illumination day and night. The IVC home cage sits immediately above a baseplate RFID reader. The mini-computer captures the video and baseplate data. The system enables manual behavioral analysis at any time of day or night, overall motion detection (whole-cage activity) for the group of rats, and automated detection of ambulatory and vertical activity, and subcutaneous temperature. Representative 7-day readouts are shown for ambulatory activity and subcutaneous temperature; the 12 h light-dark cycle is indicated by white-gray shading.

In 2011 AstraZeneca set a challenge under the National Centre for the 3Rs (NC3Rs; [51; 52]) inaugural CRACK IT open innovation scheme [53], calling for novel technology to record activity, behavior and temperature of individual rats continuously when group-housed in conventional individually ventilated home cages in a portable cage rack (the ‘Rodent Big Brother’ project). The specifications also included the requirement for a rack-based (rather than bench top) system, an important feature with regard to welfare and husbandry requirements, incorporation into long-term studies, and space constraints. We have developed home cage continuous monitoring technology (Fig 1) that can acquire ambulatory activity and subcutaneous temperature of individual rats when group-housed, for at least 28 days, 24 hours per day. The system avoids invasive surgery and the technology is unobtrusive. In addition it acquires continuous side-view high-definition (HD) video of the entire cage, which can be used to extract additional behaviors from the video. This paper describes the technological development, evaluation, optimisation and validation of the approach.

## Methods

### Description of the hardware

The Home Cage Analyser (ActualHCA™) system (Actual Analytics Ltd, UK) fits in a standard IVC cage rack (e.g., SealSafe Blue-line range, Tecniplast S.p.A., Buguggiate, Italy), with infrared lighting strips located above the cage, a baseplate beneath the cage, and a camera, computer and power supplies in a vacant cage slot to the side of the cage (Fig 1). This obviates the need for either bespoke caging or benching, and enables the rats to be group-housed in their normal home cage in an adapted IVC cage rack.

RFID dual identification and temperature transponders (factory calibrated) were supplied by BioMark/Destron (Boise, ID 83702, USA). The Biomark BioTherm13 Passive Integrated Transponder (PIT) is an RFID device that complies with the specifications of ISO Standards 11784 (ID code compatibility) and ISO 11785 (communications protocol). This PIT Tag is packaged in a laser-annealed glass ampoule that is designed specifically for subcutaneous (or intramuscular) implantation. Dimensions are 2.12 ± 0.10 mm in diameter x 13 ± 0.4 mm in length. Temperature recording range: 33.0°C to 43.0°C; accuracy ± 0.5°C (factory pre-calibrated).

For the baseplate RFID reader, Actual Analytics worked with BioMark USA to specify and design a baseplate that would work with the BioTherm 13 RFID transponders. This comprised a 2D arrangement of twelve transceiver coils (in a 3 x 4 array) in a waterproof casing. The dimensions of the casing were 38 x 50 cm, approximately the dimensions of the base of the rat cage.

**Fig 2:**
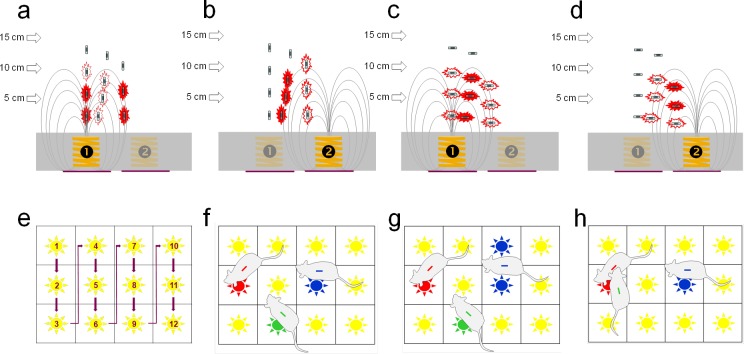
Schematic overview of RFID transponder activation and detection by baseplate. Upper panels: Side view of baseplate showing 2 adjacent antennae (‘1’ and ‘2’) with figurative magnetic field lines generated by active antenna (indicated by brighter color). The thickness of the jagged red outline around the RFID transponder indicates signal strength, generated by magnetic induction; this is maximal when aligned with the magnetic field direction. Each antenna is activated for 75 ms. (a; b) RFID transponder in horizontal orientation in various positions above the baseplate; signal strength is generally highest in between antennae. (c; d) RFID transponder in vertical orientation in various positions above the baseplate; signal strength is highest directly above an active antenna or where magnetic field lines are returning towards the baseplate in a near-vertical plane between adjacent antennae. In this way, there are no ‘dead spots’ across the baseplate. Lower panels: Birds-eye view of the baseplate. (e) Read sequence of the 12 antennae; total scan cycle takes 1080 ms. (f) Rats are detected by the nearest antenna, reading the ID and temperature from the RFID transponder. (g) At intermediate positions, an animal (blue) may be detected by two (or more) adjacent antenna. This can cause ‘flickering’ of the motion detection between the two antennae when the animal is virtually stationary, minimized by applying a basic filtering algorithm. (h) Two animals (red and green) close to the same antenna, resulting in detection of the stronger signal (red) and temporary drop-out of the RFID signal from the other individual (green).

**Fig 3:**
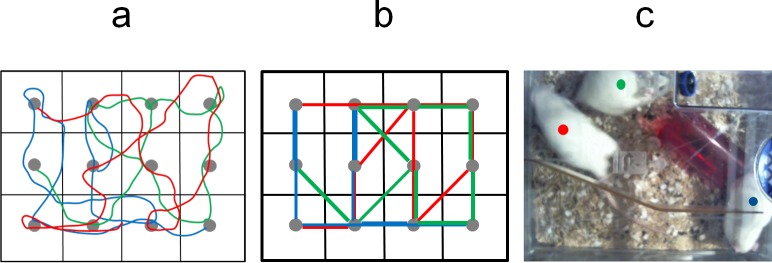
Illustration of difference between actual track of the rats and automated tracking of RFID transponder to nearest of 12 antennae in the baseplate. (a) View through transparent cage lid (food pellets and water bottle temporarily removed) via temporary birds-eye camera. Each colored dot was manually positioned once every 25 frames (i.e., once per second) on the center of each rat as they moved around over 60 min. (N.B. For the ventral midline RFID position, this is close to that position.) (b) Actual track of each rat (manually derived). (c) Baseplate-derived tracking of each rat by the 12 antennae. Note that the automated positional information is centered on each antenna and is therefore truncated. This needs to be borne in mind when assessing concordance between manual (actual) and automated tracking. Whereas increasing the number of antennae would in theory improve spatial resolution, their smaller size would reduce the vertical reach of each magnetic field, so the number used (12) was selected to achieve optimal performance in rats.

Each antenna is powered up sequentially and then the strongest RFID tag within the vicinity of the electromagnetic field is read (Fig 2). A complete cycle of the 12 antenna takes 1.1s (0.93 Hz). Note that due to the physics of the system, an RFID tag may be reported on the nearest as well as adjacent antennae, if two tags are present on an antennae, only one will be reported and finally the tag needs to be within the active detection field for sufficient time for it to be charged and read (~60 ms) therefore rapidly moving animals can be missed for a few cycles. Spatial filtering is used to resolve the most likely location given an array of reads and interpolation to estimate mostly likely positions between reads. In all cases both raw data and filtered/interpolated data are retained and marked as such. The automated ambulatory tracking will be truncated compared to the actual path taken by the animal (Fig 3).

To enable continuous collection of HD video, each module incorporates strips of infrared LEDs at 860 nm wavelength, to illuminate the cage from above. A USB 3.0 camera with matched 4.5mm lenses and daylight filters (700 nm cut-off) captures infrared (grayscale) video at 25 fps at HD (720p) resolution.

During the course of this project, the performance of the Actual HCA hardware was improved by increasing the magnetic field strength, achieved by a system upgrade that included tuning the baseplates and additional Faraday-shielding of sources of stray electromagnetic fields (notably, the rack-located power supplies). This is collectively referred to as the ‘shielding upgrade’ within this article.

### Software and architecture

#### Data capture

A small onboard computer runs a package called Actual HCA Capture™ (Actual Analytics Ltd, Edinburgh, UK), to manage the video capture, calibrate the system, and reboot the system in the event of power failures. Chunks of video (user-defined but typically 15–30 minutes) and matched baseplate data are captured and stored to a local hard drive.

#### Data analysis

A second piece of software, Actual HCA Analyser™ (Actual Analytics Ltd, Edinburgh, UK), which reads and analyses the raw data generated by Actual HCA Analyser™ can be run on any remote computer. It provides the opportunity to view the data, analyse/filter raw data and produce video overlays combining analytics and video. Raw data of movement and temperature from the RFID transponder are recorded via the baseplate as the number of transitions between baseplate antennae, and subcutaneous temperature, both tied to each individual animal. Video footage can be analysed using motion detection for cage level events (e.g., video-based motion detection) or to extract individual behaviors. The software also includes a range of analytical algorithms (e.g. activity statistics; circadian rhythm analysis; location preference; etc.).

#### Server

A third and optional software component helps manage a multi-cage installation. It automates the copying of the data files from individual units onto a server. It also automates some of the post capture data processing thereby reducing the number of processes that the Analyser in the computer of each enclosure has to do. This speeds up analysis and interaction with the data. It also manages the application of post-hoc analytics for automated behavioral detection that is then available for review on the Analyser.

### Assessing RFID transponder efficiency at different heights and orientations above the baseplate

Evaluation of the impact of the spatial orientation and height of the RFID transponders was achieved by placing an RFID transponder on an adjustable non-metallic platform on the surface of a baseplate. The device was placed above each baseplate for 60 s over a height range of 4–13 cm, in 1 cm increments, and the signal read frequency for each recording was analysed. Before and after the ‘shielding upgrade’, we compared the average read rate across 4 baseplates using 2 RFID transponders in the vertical orientation at different heights above each of the 12 antennae of each baseplate. After the ‘shielding upgrade’, an evaluation was undertaken using a single RFID transponder in different positions and orientations with respect to a pair of antennae on one baseplate, namely vertical; horizontal aligned towards the centre of an antenna (X-plane); or at right angles to this orientation (Z-axis). This was done in order to evaluate the effects of spatial positioning of the RFID transponder above and between antennae, within the range expected to occur *in vivo*.

### *In vivo* studies

#### Ethical statement

The use of animals was kept to an absolute minimum required to achieve statistical significance for validation purposes; a total of 48 rats were used for the work described in this paper. All procedures were conducted in accordance with the United Kingdom Animal (Scientific Procedures) Act 1986, approved by institutional ethical review committees (Alderley Park Animal Welfare and Ethical Review Board and Babraham Institute Animal Welfare and Ethical Review Board) and conducted under the authority of the Project Licence (40/3729 and 70/8307, respectively). All animal facilities have been approved by the United Kingdom Home Office Licensing Authority and meet all current regulations and standards of the United Kingdom.

#### Animals, housing and husbandry

Male Han Wistar rats (weight range 200–275 g; age range 7–13 weeks at start of data acquisition) were obtained from Charles River Laboratories in Margate, Kent, UK. Vendor-supplied health reports indicated that the rats were free of known viral, bacterial and parasitic pathogens. They were housed in groups of 3 in Tecniplast IVC cages (model number 1500U), in a 12 h light: dark cycle with room temperature set to 21°C (recorded range: 16.9–24.5°C), in a semi-barrier facility. The cages contained a 1–1.5 cm layer of 4 mm^3^ Aspen chip bedding together with environmental enrichment (sizzlenest nesting material, Datesand) medium Aspen brick chew sticks (Datesand) and a red plastic play tunnel (transparent in infrared light). The rats had access to food (RM1 (E) IRR 0.25 pelleted diet, Special Diet Services, UK) and water ad libitum. Rats were allocated to cages on arrival and remained in the same social group throughout the study. They were identified by waterproof tail markings. Rats were test-naïve prior to the studies. Animal welfare was assessed throughout by daily monitoring of appearance, behavior and cage environment. Rats were allowed to acclimatise to the animal unit for at least 1 week before implantation of the RFID transponder, with at least a further 2 days of recovery post-implantation before using acquired data.

#### Selection of optimal implantation site for the RFID transponder

**Fig 4:**
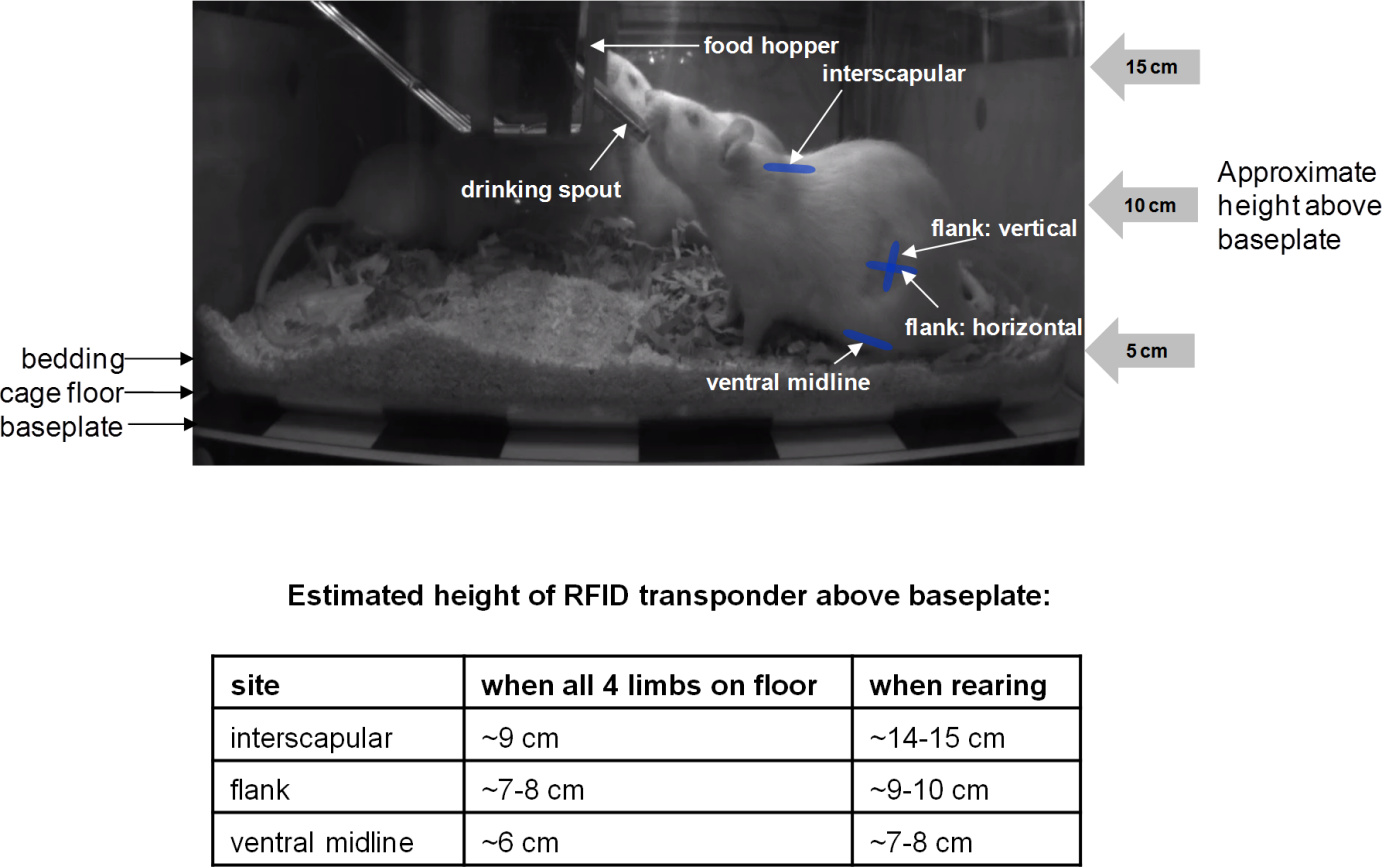
Schematic illustration of the 4 implantation sites/orientations for the RFID transponder evaluated. The ventral midline site generally remains closer to the baseplate than the other locations. Unlike the other locations, the flank location (whether flank: vertical or flank: horizontal) is offset from the midline of the rat.

Four different subcutaneous injection sites for the RFID transponder were compared: interscapular, flank: vertical orientation, flank: horizontal orientation, and ventral midline (‘vertical’ and ‘horizontal’ refer to the approximate orientation of the RFID transponder with the rat standing on all four limbs; Fig 4). In the initial optimisation study, each rat was implanted with one transponder under brief anaesthesia with isoflurane (induced at 4.5%, maintained at 3.0–4.0%) during implantation. The experiment was undertaken over a time period of several weeks, in 3 cohorts of rats, n = 6 (i.e., 2 cages) per implantation site. As there was no other prior information a group size of 6 males was chosen, which is commonly used in the Irwin test/FOB [7;10]. A follow-up study was undertaken comparing the two best sites evident from the initial comparison study (ventral midline and flank: vertical), with two sets of 6 rats in a direct, head-to-head comparison of these two sites, following improvement of baseplate performance by the ‘shielding upgrade’. For the ventral midline site there is limited loose skin and care has to be taken not to inject the transponder intraperitoneally; creating a small subcutaneous pocket prior to inserting the pre-sterilised trocar needle, ejecting the RFID transponder, and sealing the entry site with surgical adhesive was preferred.

The performance/experience of each site was compared across the following pre-set criteria (Table 1): ease of implantation; lack of inadvertent intraperitoneal injection/migration from original injection site/external extrusion; RFID signal strength (read frequency); baseplate individual ambulatory activity tracking vs. birds-eye individual manual tracking (for 60 min, achieved by mounting a video camera pointing vertically downwards through the transparent cage roof; Fig 3); baseplate individual ambulatory activity tracking vs. side view motion detection (2 cages of 3 rats; 24 h and 3–4 weeks); light-dark phase ambulatory activity contrast; light-dark phase temperature contrast; histopathology of implantation site to detect any evidence of inflammation.

**Table 1:** Criteria used to select optimal subcutaneous RFID implantation site/orientation.

Criterion	Scoring system applied
Relative ease of implantation	Subjective score of 1–5 based on convenience, speed, whether it required an assistant; accidental intraperitoneal implantation.
Lack of inadvertent intraperitoneal (i.p.) injection/migration from original injection site/external extrusion	Detected post mortem; score of 5. no issues; 4. inadvertent i.p. injection/migration in 1/6 rats; 3. in 2/6 rats
Read frequency	Recorded by the software; reflects ‘signal drop-out’. Maximum theoretical performance is a read frequency of 0.93 Hz. Scores of 5. >0.75 Hz; 4. >0.6<0.75 Hz; 3. >0.4<0.6 Hz; 2. >0.3<0.4 Hz; 1. <0.3 Hz
Baseplate individual ambulatory activity tracking vs. birds-eye individual manual tracking (1 h)	Each cage of 3 rats viewed from above (‘birds-eye view’) through the transparent cage lid for 60 min using a webcam. Each rat tracked manually, and compared to baseline ambulatory activity. Scores of 5. ICC >0.9; 4. ICC >0.7<0.9; 3. ICC >0.5<0.7; 2. ICC >0<0.5; 1. ICC <0.
Baseplate individual ambulatory activity tracking vs. side view video motion detection (cage of 3 rats; 24 h and 3–4 weeks)	Each cage of 3 rats viewed side-on for 24 h via the integral HD camera. Ambulatory activity of each individual rat within a cage of 3 compared to the group overall activity, derived from video motion analysis. Scores of 5. R^2^ >0.9; 4. R^2^ >0.7<0.9; 3. R^2^ >0.5<0.7; 2. R^2^ >0.3<0.5; 1 R^2^ <0.3.
Histopathology of implantation site	Evaluation of any inflammatory response (none was observed at any site).

ICC = intraclass correlation.

#### Validation of vertical activity measurements

**Fig 5:**
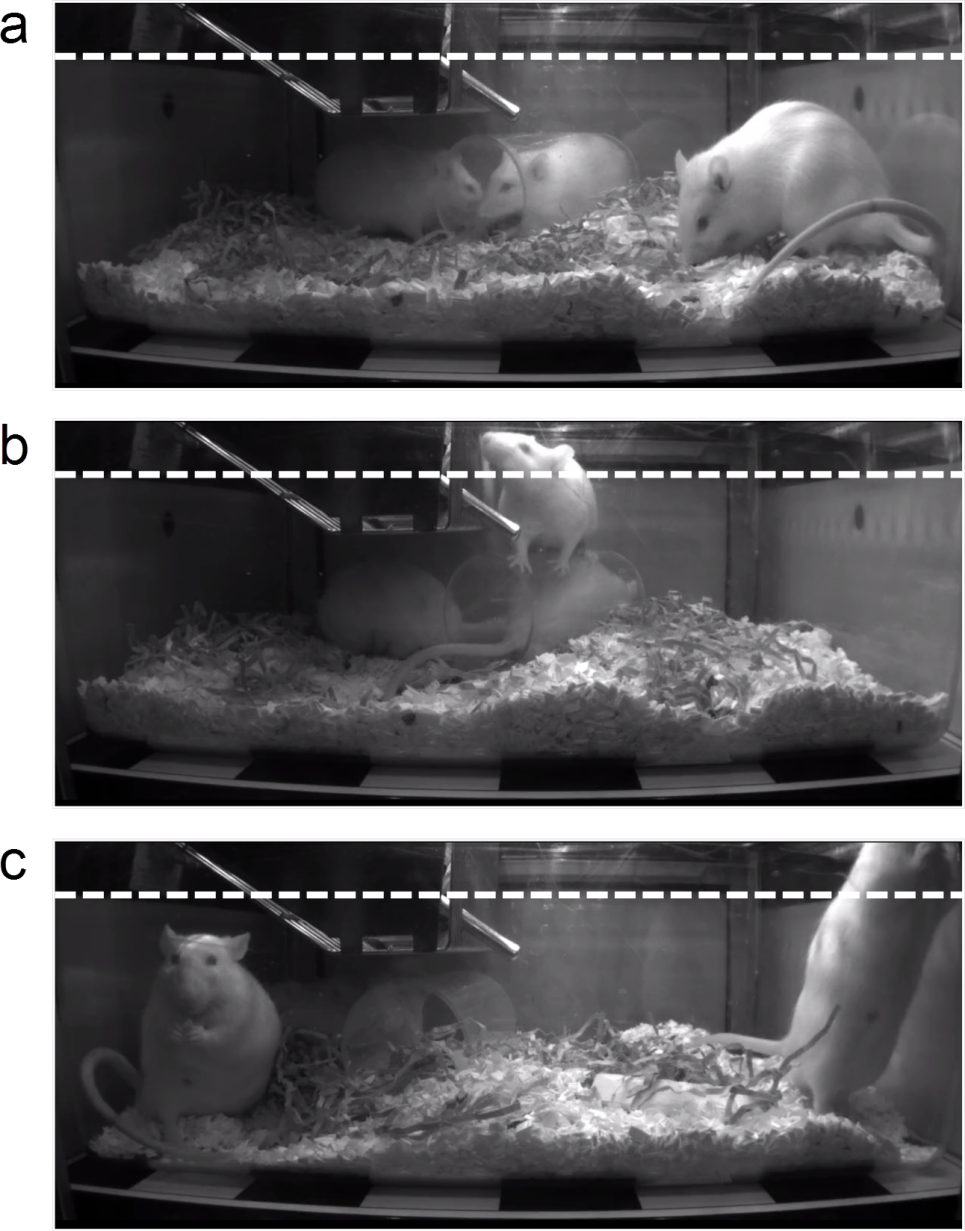
Assessing the precision of the vertical activity measure. (a-c) Still images from side-mounted HD camera showing position of arbitrary cut-off line to define ‘vertical activity’ (image movement detected above dotted line). (a) No vertical activity, no rearing; (b) vertical activity not due to rearing (rat in center of image is climbing on plastic play tunnel); (c) vertical activity due to rearing (rat on right of image; the rat on the left of the image is eating from its forepaws. Note that the automated detection of vertical activity is not exactly equivalent to rearing behavior, as vertical activity also includes climbing on the play tunnel and (particularly when rats are in the foreground of the image) tail elevation, transient postural elevation during shaking during grooming, and jumping on cagemates during play fighting. Conversely, some rearing in the background of the image is partly obscured by the food hopper or may not quite reach the level of the cut-off line.

This was undertaken on video footage from the above studies. Male Han Wistar rats were recorded continuously for 7 consecutive days (frame rate set at 25 frames per second) when housed in groups of 3 in standard individually ventilated cages. Episodes of vertical activity, defined as any movement above a vertical threshold (set at 8 cm at the far wall of the cage), and rearing behaviour (when front paws were elevated and rat was in an upright posture) were manually annotated using ActualTrack™ on two 1-hour samples from the light phase and the dark phase, respectively (Fig 5).

The automated detection of vertical activity per cage was correlated against manual annotation of vertical activity and against the more strictly defined rearing behaviour for each cage from both light and dark phase video footage. The accuracy between the automated detection and manual annotations was determined by frame-by-frame accuracy over each 15-min bin from the light and dark phase video footage. The vertical activity of individual rats in each cage was also identified for the automated detection using the individually implanted subcutaneous RFID transponder. Episodes of rearing behaviour of individual rats in each cage were manually counted from the light and dark phase video footage, to compare with the automated detection.

### Initial *in vivo* observations using the new technology

Side-view motion detection (pixel movement) was undertaken on continuous 7-day HD video from 12 separate cages, each containing 3 rats, tested in the same facility spanning an 8-month period. This comprised all the rats used in the present study. Although they had RFID transponders in different subcutaneous locations, this was not relevant to the video analysis. The 12-hour activities for light phase and for dark phase were averaged separately over a 7-day period.

Two environmental changes were introduced (separately) in order to evaluate whether the ActualHCA™ system could detect perturbations: single-housing rats for 16 h (to assess effects on subcutaneous temperature), and cage changing (to assess effects on ambulatory activity). Cage changing occurs once per week for laboratory rats in our facility, and comprises relocation of the social group of 3 rats into a clean cage with fresh bedding and environmental enrichment. This is known to stimulate activity temporarily as the animals explore their new surroundings. The effects of a dosing procedure (oral gavage with water, 10 mL/kg) was also evaluated.

### Terminal procedures and histopathology

All animals were euthanized by overdose of isoflurane in accordance with the Humane Killing of Animals under Schedule 1 to the Animals (Scientific Procedures) Act 1986. The subcutaneous implantation sites were grossly examined for abnormalities and thereafter collected including the RFID microchip in-situ. Tissues around the implantation site were fixed in 10% neutral buffered formalin. All tissues were processed to wax blocks, sectioned, stained with hematoxylin & eosin (H&E), and examined microscopically.

### Statistical analysis

#### Assessing RFID transponder efficiency at different orientations

A linear regression analysis, fitting Eq 1, was used to explore the variation in the read rate from the RFID transponder when a range of elevations and orientations were tested across a number of different baseplates. The model was developed based on domain knowledge, following graphical exploration of data. An interaction between baseplate*shielded was considered but not found to be significant (data not shown). Terms were selected as significant in explaining variation at p<0.05 threshold. Graphically the behaviour of the residuals were explored to assess the quality of the model fit and found the model was a good fit to the data (data not shown).

readrate=shielded+height+RFID.ID+baseplate [Eq 1]

A mixed model regression analysis, fitting Eq 2, was used to explore the variation in the read rate from the RFID transponder fitted *in vivo* for a range of positions within the animals. Implantation site, baseplate and whether the instrument was shielded were treated as fixed effect and rat was treated as a random factor to account for the repeat readings taken from an individual animal. Model optimisation included a test of the covariance structure, comparing either homogenous or heterogeneous variance across implant site using a likelihood ratio test. Terms were selected as significant in explaining the variation at p<0.05 threshold. Graphically the behaviour of the residuals were explored to assess the quality of the model fit and found the model was a good fit to the data (data not shown).

readrate=shielded+baseplate+implantsite+1 [Eq 2]

#### Stability of RFID transponder performance

A two-sided paired t-test was used to compare the read rate average readings from week 1 to week 4. To ensure the statistical test was appropriate for the data, the distribution of the difference was explored graphically and found to be normally distributed.

#### Validation of measure

To evaluate agreement between two approaches to a measure a Bland-Altman plot [54] was used to analyses the distribution of the differences between measures. The intraclass correlation coefficient (ICC)(method: ICC1k average absolute agreement) [55] was calculated as a measure of agreement.

#### Sources of variation in temperature measure

A mixed model regression analysis, fitting Eq 3, was used to explore the variation in the temperature reading for data collected over four weeks. Terms were selected as significant in explaining the variation at p<0.05 threshold. Graphically the behaviour of the residuals were explored to assess the quality of the model fit and found the model was a good fit to the data (data not shown).

dependentvariable=day+cage+week+baseplate+phase+1|Rat [Eq 3]

#### Sources of variation in number of transitions measure

A mixed model regression analysis, fitting Eq 3, was used to explore the variation in the number of transitions when summed in 15 minute bins for data collected over four weeks. Terms were selected as significant in explaining the variation at p<0.05 threshold. Graphically the behaviour of the residuals was explored to assess the quality of the model fit (data not shown) and the model had issues with the data being bound due to a high presences of zeros where the animal did not move. An alternative Poisson model was not suitable due to over-dispersion. A negative binomial model was fitted and diagnostics were good, providing the same conclusions as the mixed model regression. The standard regression results are presented within the manuscript as they are easier to interpret.

Effects of cage changing on activity and temperature and the effects of single housing on subcutaneous temperature were not evaluated by statistical analysis, as these were casual observations rather than pre-designed experiments.

Data are expressed as mean ± standard error of the mean (SEM) unless otherwise stated.

### Data availability

All data generated or analysed during this study are included in this published article or are deposited in Zenodo: 10.5281/zenodo.804041

## Results

### Assessing RFID transponder efficiency in different spatial orientations

**Fig 6:**
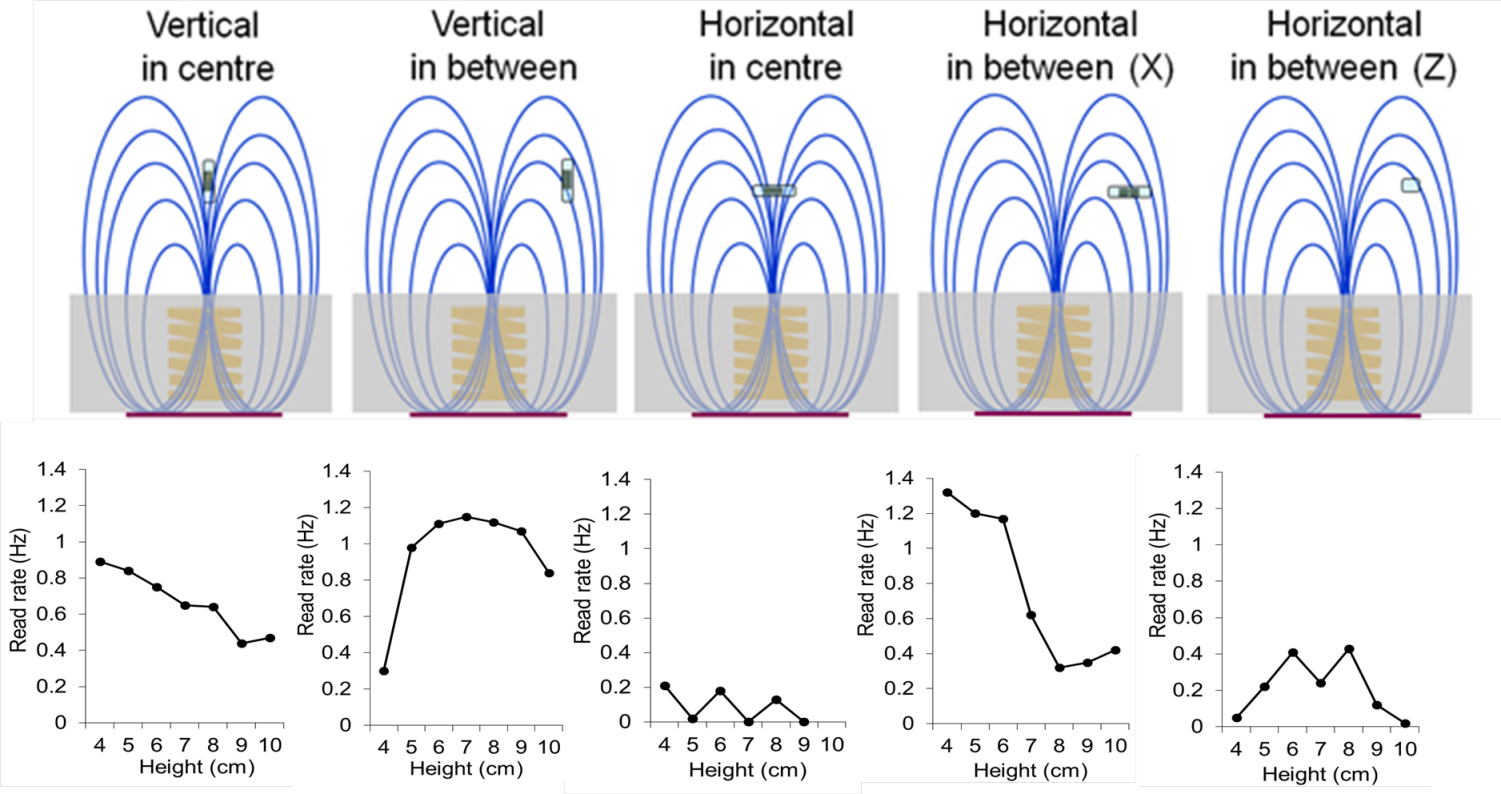
Impact of height and position on RFID transponder read rate. Mapping of signal strengths (as ‘read rates’) with an RFID transponder at different orientations, positions and heights with respect to a baseplate antenna. Upper panel: Diagram of magnetic fields of an active antenna and the orientation of the RFID transponder. ‘X’ and ‘Z’ indicate two planes of horizontal orientation, with the RFID transponder pointing towards the center of the antenna (X orientation) or at right angles to it (Z orientation) Lower panel: Read rate analysis for different orientations, positions and heights above the baseplate. These data underlie the schematic representation in Fig 2A–2D.

*‘Ex vivo’*, we explored a range of elevations/orientations of the RFID transponder above and around different baseplates, *in situ* in the cage rack in the animal housing room. This enabled us to map the RFID signal strengths, which result from the interplay between the strength and direction of the magnetic fields generated by the baseplate antennae, and the orientation and height of the RFID transponder, across the baseplates (Figs 2 and 5). Fig 6 indicates that the read frequency (‘read rate’) will vary as an animal moves across the baseplate, or rears/climbs. A regression analysis assessing for sources of variation of read rate found only two variables apart from height that were statistically significant (S1 and S2 Figs), namely the ‘shielding upgrade’ and inter-baseplate variability. These were statistically significant but only had a small effect on read rate: the ‘shielding upgrade’ increased the read rate (by 0.06 ± 0.01 Hz; P = 0.0000125) and one of the baseplates had a tendency to lower read rates (by 0.052 ± 0.02 Hz; P = 0.012).

**Fig 7:**
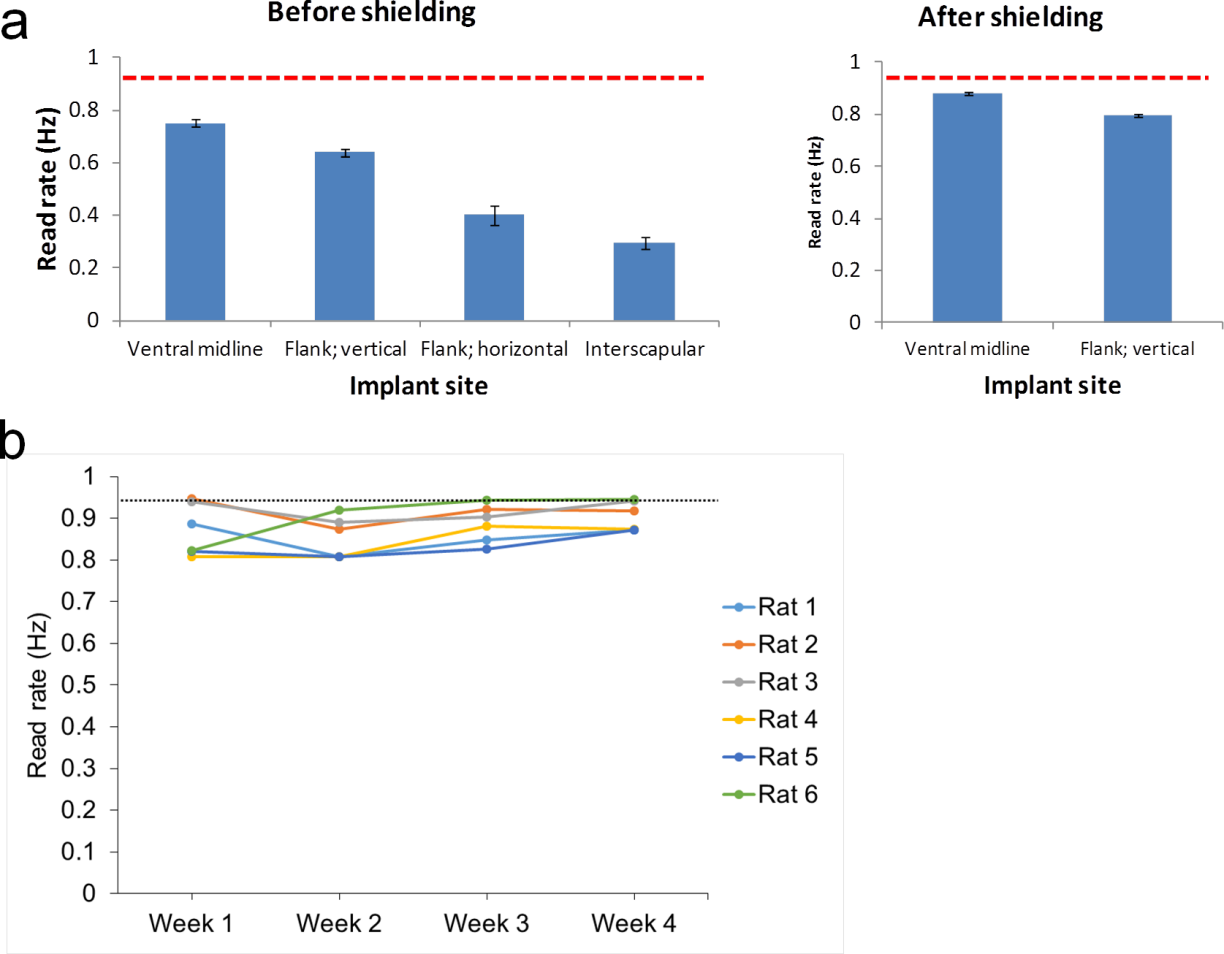
RFID read frequencies from the 4 implantation sites, improvement by baseplate ‘shielding upgrade’, and maintenance of RFID performance over 4 weeks of continuous *in vivo* use. (a) Mean (± SEM) RFID read frequency when implanted in rats over 28 days, before and after enhancement to antenna magnetic field strengths by Faraday-shielding power supplies in the vicinity and tuning of the baseplates (‘shielding upgrade’). Left-hand panel: Initial comparison of the 4 RFID implantation sites, conducted over 3 cohorts of rats spaced across several weeks. Each column is the mean read frequency over a 7-day period (n = 5 rats for ventral midline implantation site; n = 6 for the other sites). Right-hand panel: Head-to-head comparison of flank: vertical and ventral midline sites, following the ‘shielding upgrade’ (n = 6 rats for both implant sites). The data indicate that the performance ranking was ventral midline > flank: vertical >> flank: horizontal > interscapular. Read frequencies for ventral midline are close to the theoretical maximum (0.93 Hz), indicated by the red dotted line. (b) Stability of RFID read frequency implanted in rats over 4 weeks (ventral midline location; post-‘shielding upgrade’). Each column is the mean read frequency over a 7-day period for each of the 6 rats. The data indicate that the read frequencies are close to the theoretical maximum (0.93 Hz; red dotted line) and that there is no drop-off in read frequency over 4 weeks of continuous use, indicating that there is no loss of performance of the RFID transponders (or antennae/receivers).

*In vivo*, four different locations/orientations were evaluated for subcutaneous implantation of the RFID transponder, namely interscapular, flank: vertical, flank: horizontal, and ventral midline (6 rats per implantation site) (Fig 4). A regression analysis assessing for sources of variation within the read rate *in vivo* found that differences between individual baseplates had a statistically significant but minor impact (considered to be within normal manufacturing tolerances) whilst the ‘shielding upgrade’ increased the read rate considerably (by 0.14 ± 0.02 Hz; Fig 7A and S3 Fig). The impact of transponder position was even more substantial with the best read rate seen with the RFID transponder implanted in the ventral midline position (0.74 ± 0.02 Hz, pre-‘shielding upgrade’). Compared to the ventral midline position, the read frequency from the flank: vertical site was on average 0.1 ± 0.02 Hz lower, the flank: horizontal site was 0.3 ± 0.04 Hz lower and the interscapular site was 0.44 ± 0.03 Hz lower. The ‘shielding upgrade’ improved the read rate for ventral midline (and flank: vertical), as illustrated in Fig 7A.

### Stability of performance of RFID transponders over time

*In vivo*, read frequency of the RFID transponders remained constant throughout the 4 weeks of continuous testing (Fig 7B). This was reassuring, as they are being activated ~2.5 million times over a 28-day period.

### Post-mortem histological evaluation of RFID implantation sites

**Fig 8:**
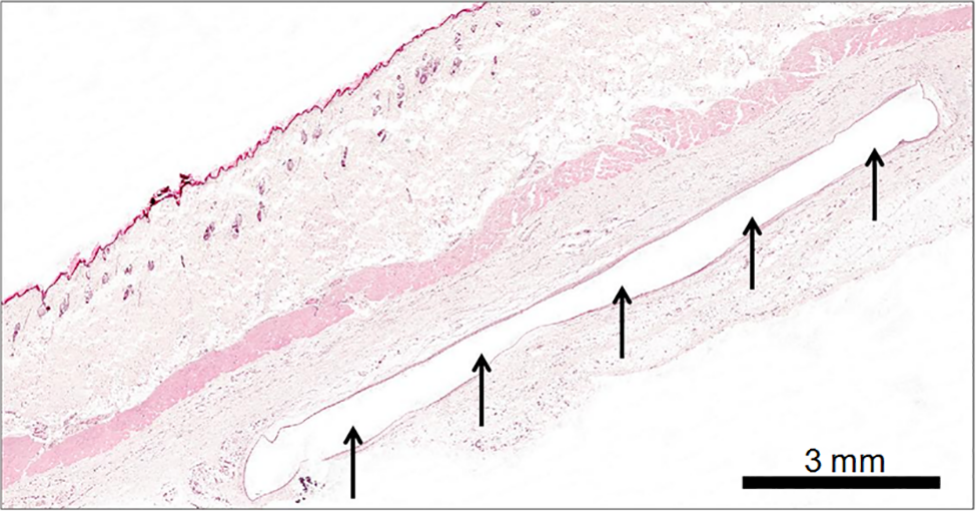
Evidence that the RFID transponders do not elicit an inflammatory tissue reaction. Subcutaneous empty pocket (arrowed) displays site of former localisation of RFID microchip. No or slight cellular reaction (focal chronic reactive granulation tissue) was observed histologically after H&E staining of skin collected from the area of implantation.

The skin and surrounding tissues around the implanted microchip showed no gross abnormalities. Subcutaneous empty pouches were indicative of the former localisation of the RFID microchip. No or slightest cellular reactions (minimal focal chronic reactive granulation tissue) were observed microscopically in H&E stained sections collected from the implantation area of all rats. Fig 8 is a typical illustration of this finding. Histopathologically, no clear preferred implant site could be identified.

### Validation of the automated ambulatory activity measure

**Fig 9:**
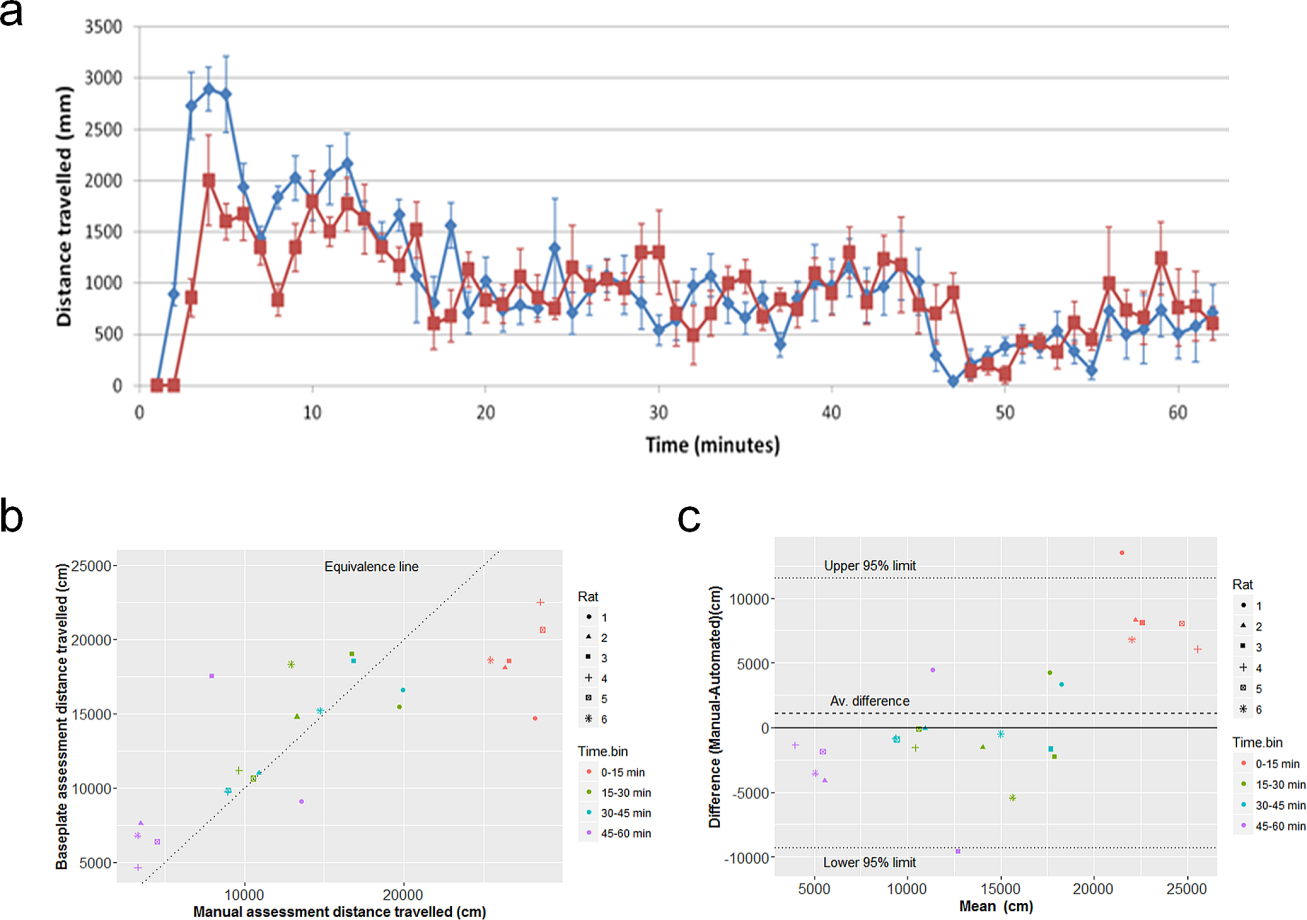
Validation of baseplate-derived ambulatory activity by comparison to manual tracking. Data shown are from the ventral midline RFID placement following ‘shielding upgrade’. (a) Ambulatory movement (distance travelled) of the rats derived from the baseplate RFID reader (red line) is overlaid with distance travelled measured by manual tracking (blue line) over a 60 min period (plotted in 1-minute bins; mean of 6 rats ± SEM), for each RFID implantation site. (b) Correlation plot of baseplate and manual activity data (distance travelled) from the 6 rats plotted in 15-minute bins from the 60-minute monitoring period. The dotted line is the equivalence line. (c) Bland-Altman plot showing the average difference between the baseplate and manual measurements as a function of the average reading.

This was addressed in two ways, for each of the 4 RFID implantation sites (n = 5–6 per site): by comparison to manual tracking via a birds-eye view camera over a 60-minute period, and by comparison to side-view motion detection over 7–28 days. A correlation plot and a Bland-Altman plot of the automated readout against the birds-eye manual tracking found the quality of the correlation depended on the RFID implantation site (S6–S9 Figs). The correlation was highest for the ventral midline site (ICC = 0.83); visual inspection of the correlation plot highlighted a drop in the automated measure below the line of equivalence at higher levels of activity (Fig 9). As evident in the time course plot (Fig 9A), the highest levels of activity occurred during the initial part of the 60-minute assessment (immediately following the disturbance to the animals caused by placing the cage in the rack). It is likely there was more thigmotaxis accompanied by rearing during the first few minutes which together with faster locomotion will tend to impact on tracking accuracy.

**Fig 10:**
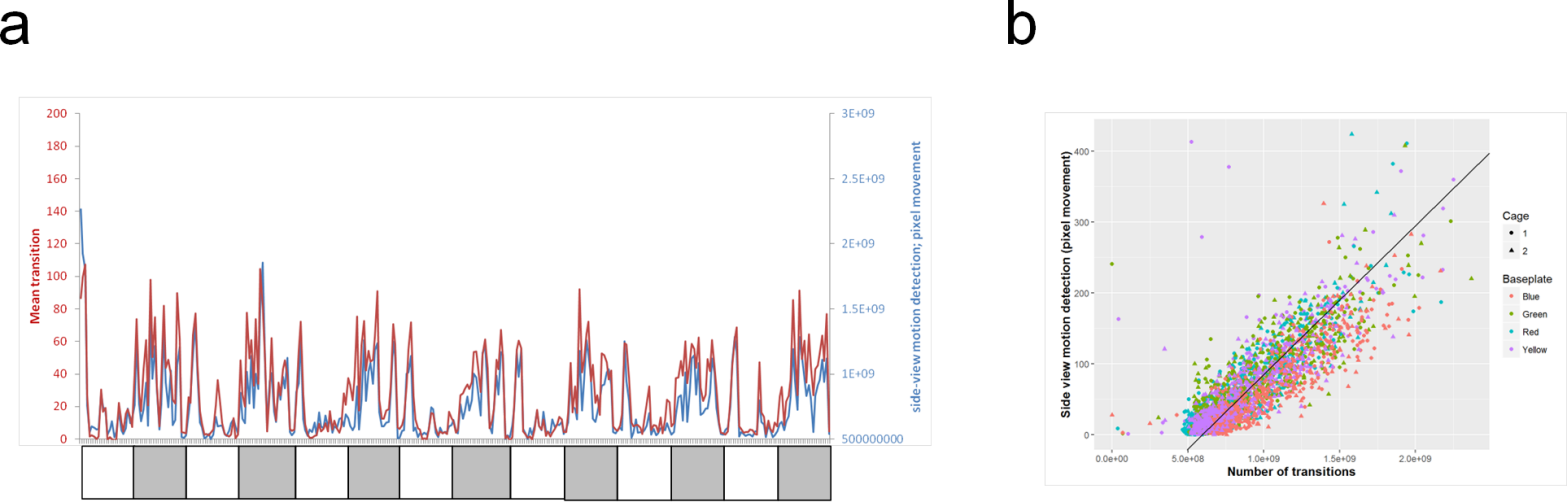
Concordance between baseplate-derived ambulatory activity and overall movement within the cage by video analysis. Overlay plot from one cage of 3 rats (a) and correlation plot for 2 cages of 3 rats (b) of ambulatory movement of the rats derived from the baseplate RFID reader versus side-view pixel movement detection, for the ventral midline site (after ‘shielding upgrade’), over 7 consecutive days of recording. Data are plotted as the mean of 3 rats per cage (2 cages), in 30-minute bins. For the overlay plot (a), the red line is mean transitions derived from the baseplate, the blue line is the total pixel movement derived from the side-view HD video; light and dark phases are indicated by the shading. Note that the video motion analysis reflects all movement (not just ambulatory activity), and may at times be exaggerated by a rat in the foreground grooming (for example).

Similarly, concordance between the baseplate-derived ambulatory activity and the side-view, whole-cage pixel movement was also dependent on the RFID implantation site (S10 Fig). The correlation was highest for the ventral midline and flank: vertical sites. Fig 10 illustrates this concordance for the ventral midline RFID implantation site (post-‘shielding upgrade’) over 7 days). The concordance is good despite the fact that the latter measurement also includes non-ambulatory movements.

### Exploring sources of variation in the raw data

Regression analysis was used to assess what factors were adding variation to the ambulatory activity measure (a function based on transitions detected) when the data was summed in 15 minute bins *in vivo* using the data from the ventral midline implantation site following the ‘shielding upgrade’ (S4 Fig). This analysis found we could detect the 15 transition decrease in activity events detected in the light phase compared to the dark phase and that day 1 and week 1 were typically 5 transitions higher than later time periods. The baseplates varied by less than 5 transitions with respect to each other within a 15 minute bin. This evaluation indicates that the technology is capable of detecting changes of this magnitude (> ~5 transitions per 15 minutes).

Regression analysis was used to assess what factors were adding variation to the temperature data obtained when data averaged across 15 minute bins *in vivo* using the ventral midline implantation site data following the ‘shielding upgrade’ (S5 Fig). The analysis found: temperatures were on average 0.25°C lower during the light phase, Day 1 has a higher temperature by around 0.05°C compared to other days, week 1 by around 0.1°C higher compared to other weeks and the ‘red’ and ‘yellow’ baseplates gave 0.05°C and 0.08°C lower readings respectively. With such a large dataset, the regression has high sensitivity to detect small changes so whilst statistically significant these are below the level of biological interest. This evaluation indicates that the technology is capable of detecting changes of this magnitude (> ~0.1°C).

### Selection of optimal implantation site for the RFID transponder

**Fig 11:**
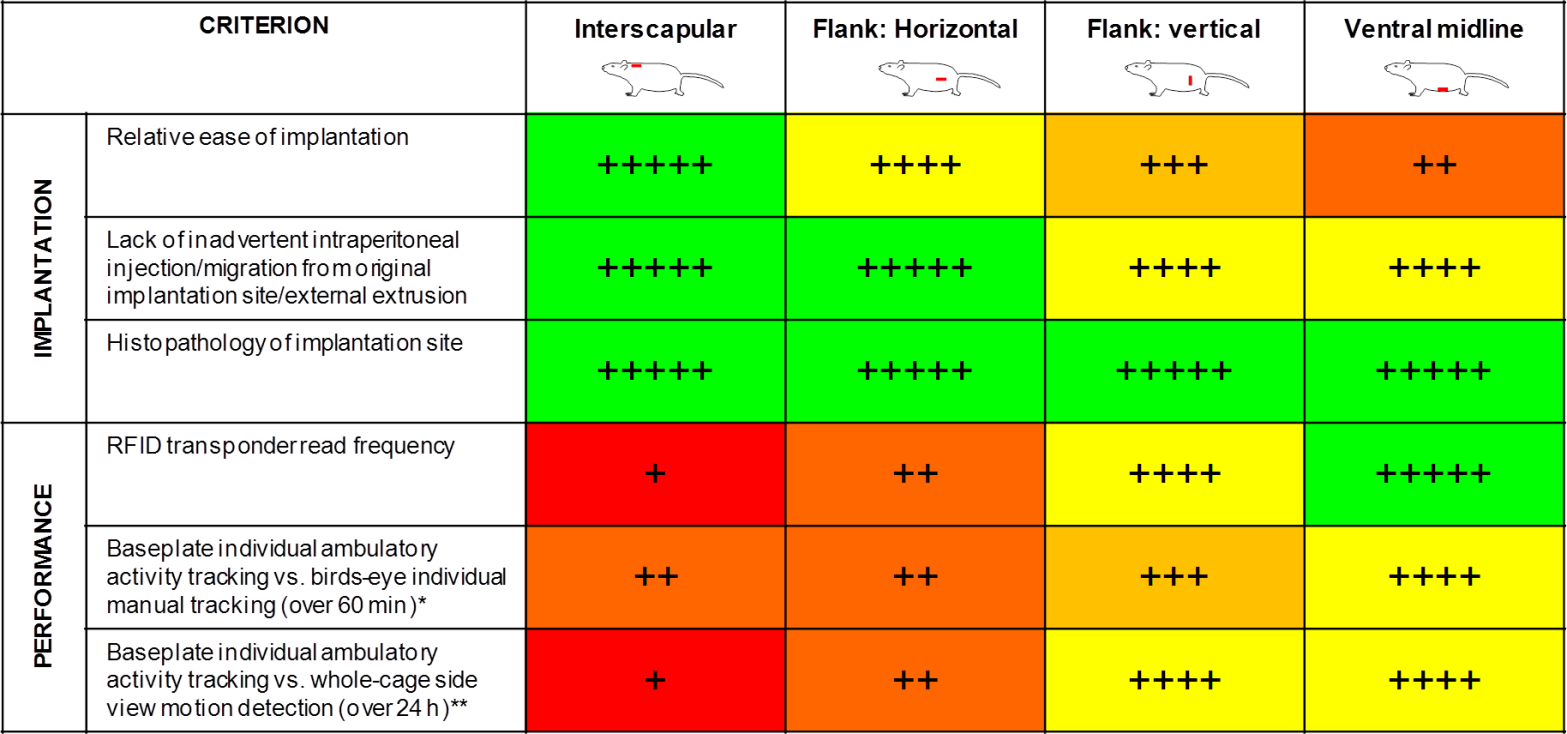
Comparison of the 4 subcutaneous RFID transponder implantation sites/ orientations. Guide to rating: +++++ (green): excellent; ++++ (yellow): optimal; +++ (light brown): near-optimal; ++ (amber): sub-optimal; + (red): poor (refer to Table 1 for rationale). *Data for flank: vertical and ventral midline are post-‘shielding upgrade’. **Baseplate-derived ambulatory activity data for the 3 individual rats within a cage averaged to compare to whole-cage activity. On balance, the ventral midline location was deemed preferable.

Four implantation sites have been evaluated and Fig 11 provides an overall comparison against the criteria set out in Table 1. The conventional and obvious location for an RFID transponder in the rat would normally be the interscapular region, as there is an abundance of loose skin. However, this location performed least well out of the four implantation sites evaluated, presumably because of the greater average height above the baseplate in this location compared to the other sites. Of four potential implantation sites, the ventral midline position was slightly superior (to flank: vertical) for recording ambulatory activity. Although a vertical orientation of the RFID transponder is optimal when directly above an antenna, where the magnetic field direction is also near-vertical (Figs 2 and 4), in between adjacent antennae a horizontal orientation is more favourable, so there is no clear advantage to either site. However, the ventral midline location is closer to the baseplate, where the magnetic field is stronger. Moreover, the flank position is offset from the midline of the animal, so that the baseplate’s position estimate is also offset from its center of mass. The only slight drawback to the ventral midline location was that it required more surgical care in the implantation (refer to Methods).

### Validating the automated vertical activity measure

**Fig 12:**
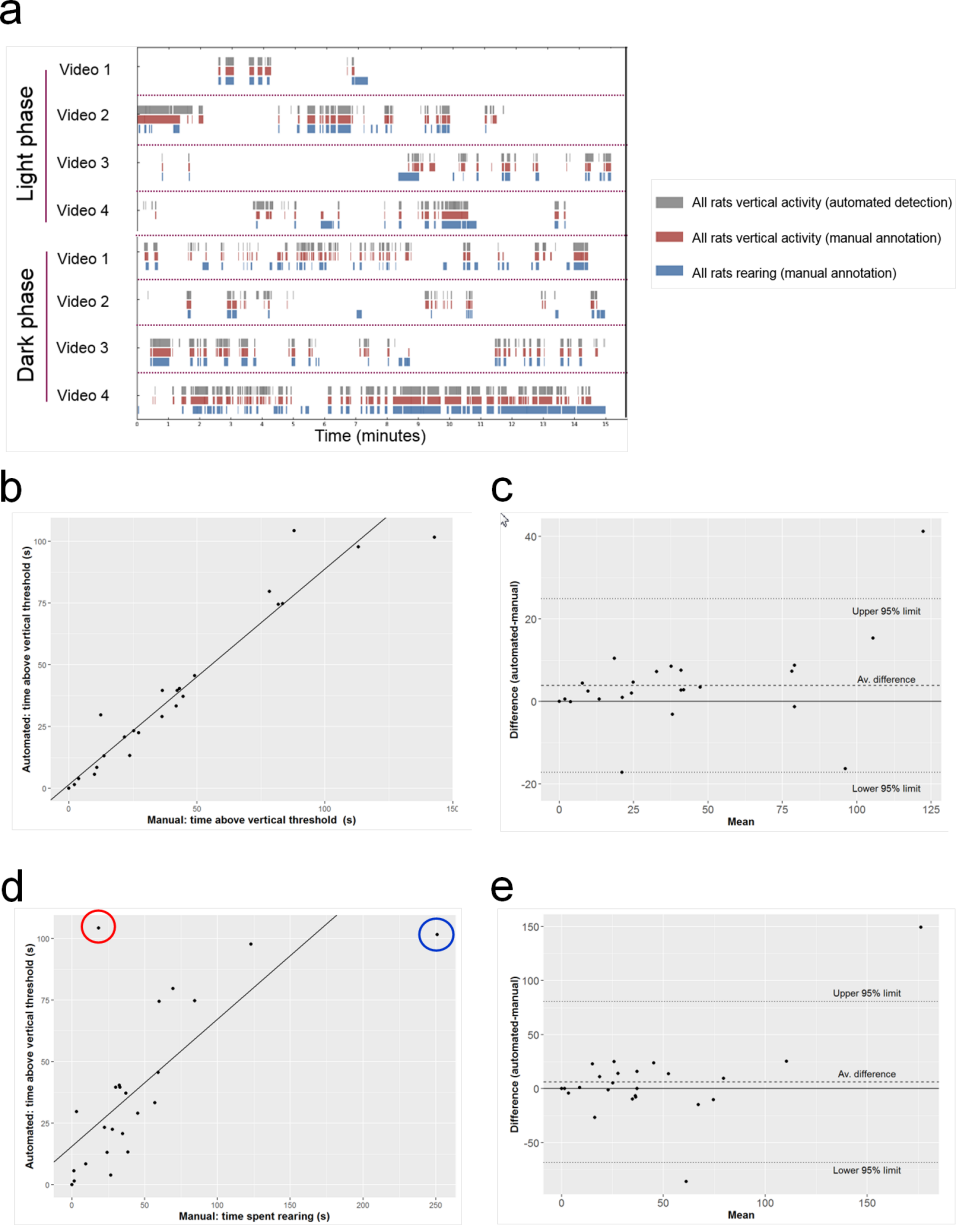
Validation of vertical activity and rearing measurements by temporal correlation. (a) Broken column charts showing the automated detection of vertical activity and manual analyses of vertical activity and rearing, in 1 h samples of light and dark phase video footage, arranged in sequential 15-min bins (videos 1–4). Gray columns indicate the time spent in vertical activity by automated detection, red columns indicate the time spent in vertical activity by manual annotation, blue columns indicate the time spent rearing by manual annotation. The frame-by-frame accuracy against the gray column of each bin was determined. (b) Correlation between time spent in vertical activity detected automatically versus manual annotation of vertical activity; R^2^ = 0.9236; ICC = 0.97. (c) A Bland-Altman plot, a visual tool to compare two techniques by plotting the difference against the signal to assess for bias in any one technique over the dynamic range assessed, was used to compare a manual measure of vertical activity and an automated measure of vertical activity. (d) Correlation between time spent in vertical activity detected automatically versus manual annotation of rearing; R^2^ = 0.4774; ICC = 0.91. Two notable outliers are highlighted; red-encircled point: probable cause is rat on top of play tunnel but not actually rearing; blue-encircled point: rat rearing at rear of cage but nose just beneath horizontal line cut-off (illustrated in Fig 5). (e) Bland-Altman plot comparing vertical measure between manual assessment of rearing and an automated assess of vertical activity.

Vertical activity was detected using automated detection of motion above a pre-set height in the cage image (Fig 5), analogous to a conventional photocell beam-break system [15;17;18;19;20]. Evaluation of two 1-hour video samples from the light and dark phases, respectively, revealed that the automated detection of vertical activity correlated well with the manual annotation of vertical activity (Fig 12). This high concordance was evidenced by an inter-class correlation of >0.91 and an overall mean frame-by-frame accuracy of 88.6 (for manual annotation of rearing) and 94.7% (for manual annotation of vertical activity). The discrepancies between automated detection and manually annotated rearing are due to behaviours that cross the vertical threshold without animals rearing (e.g., climbing on the play tunnel, tail flicking and play fighting), and the partly obscured view of the far wall of the cage due to the food hopper. Whilst we use the term ‘vertical activity’ as it is closest in definition to the actual measurements obtained, it is a very good proxy for ‘rearing’. Automated detection was also able to assign individual (rat) identity to the vertical activity events occurring within the social group, from the video footage (S11 Fig).

### Initial observations using the new technology

#### Light-dark phase differences

**Fig 13:**
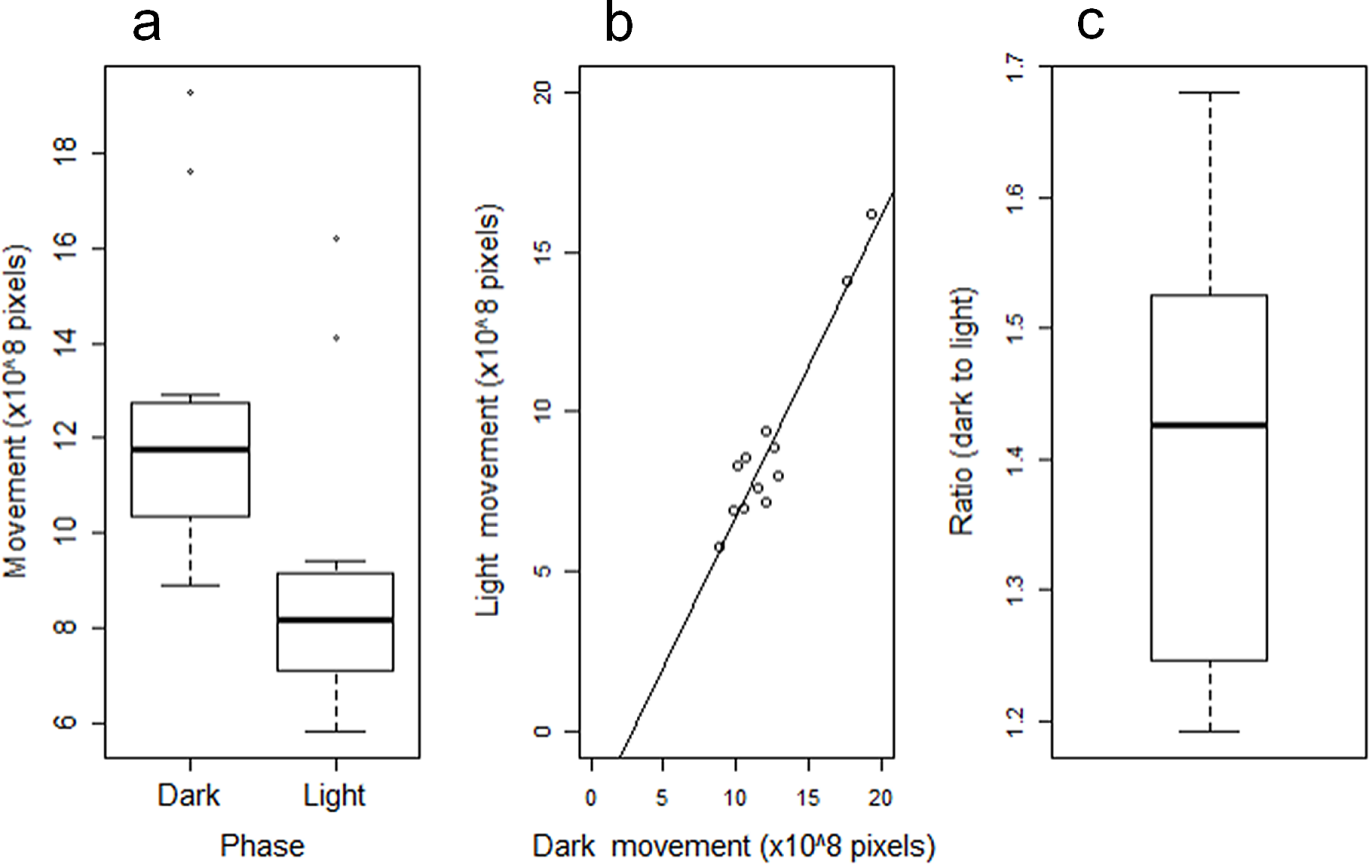
Relationship between light and dark phase overall activity for 12 cages of 3 rats. Data are 7-day mean values for the light phase overall activity (average of each of the 24 30-minute bins of side-view video pixel movement) and dark phase overall activity (ditto) for 12 cages of 3 rats. (a) Box-and-whisker plot illustrating the difference between dark and light phase activity. (b) Correlation plot between light and dark phase activity for each cage. Note that for 3 of the groups of 3 rats, their light phase activity exceeded that of the dark phase activity for at least one of the other cages. (c) Box-and-whisker plot of the ratio of dark: light phase activity. Note that although there is a range of activities between cages of 3 rats (panel b), the ratio of dark: light phase activity remains within the range 1.2 to 1.7-fold, with a mean of approximately 1.4-fold (panel c).

Using side-view motion detection on 12 separate cages, each containing 3 rats, tested in the same facility spanning an 8-month period, the 12-hour activity for light phase and for dark phase were averaged separately over a 7-day period. The range of activity levels observed were: light phase, 5.8 to 16.2 x10^8^ pixel movements; dark phase, 8.9 to 19.3 x10^8^ pixel movements (Fig 13), indicating that some groups of 3 co-housed rats were more active during the light phase than others were during their dark phase. However, the light and dark phase activities were inter-related, whereby the ratio of dark phase: light phase activity levels remained within a reasonably narrow range. The mean ratio of dark phase: light phase activity was 1.4-fold (range 1.2 to 1.7-fold). A similar ratio was found for baseplate-derived ambulatory activity data (ventral midline site) derived from 2 cages of 3 rats (i.e., n = 6), the mean ratio of dark phase: light phase activity was 1.8-fold. Fig 1 incorporates a 7-day plot of ambulatory activity, as well as temperature, illustrating a clear circadian rhythm for both ambulatory activity (see above) and subcutaneous temperature (approximately 0.5°C higher during the dark phase).

Previous publications on 24 h activity in male Wistar rats have used single-housed animals monitored by PIR sensors [21] or infrared photocell arrays [56;57], and reported that dark phase activity was approximately 4 times higher than that during the light phase. It is possible that single-housing alters behavior, augmenting the contrast in activity between dark and light phases. This would require further evaluation.

#### Effects of routine perturbations

**Fig 14:**
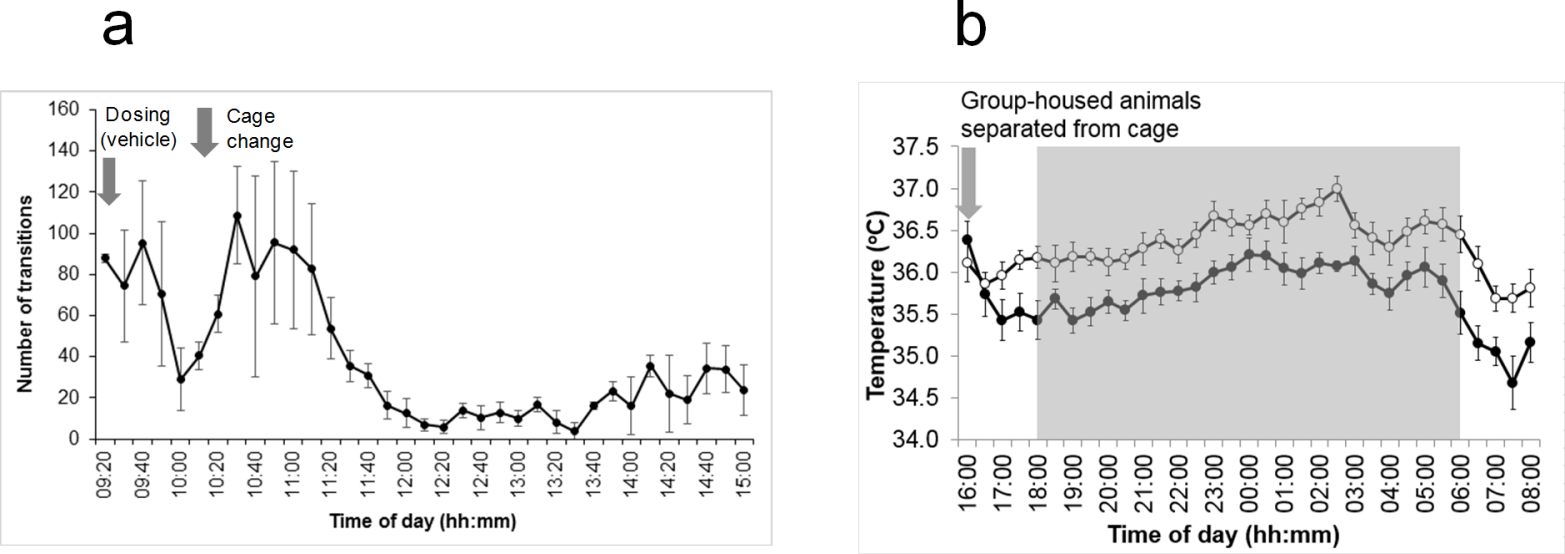
Illustration of the effects of routine perturbations on ambulatory activity and temperature. (a) Effects of handling/dosing, cage changing and entry of staff into the holding room, on ambulatory activity in the home cage. Plot of baseplate-derived ambulatory activity (mean of 6 rats ± SEM). Data plotted in 10-minute bins. The baseplate detected a spike in activity lasting around an hour in response to the change in home cage. An earlier, smaller peak was in response to dosing (oral gavage) with vehicle. (b) Subcutaneous (flank) temperature of rats when single-housed (filled circles) vs. group-housed (open circles) throughout the dark phase (gray-shaded). Data plotted in 30-minute bins (mean of 6 rats ± SEM). Rats were single-housed at 16:00 hrs and re-grouped at 08:00 hrs the following day. There was an apparent reduction in subcutaneous temperature from 1 h after separation from cage mates and throughout the 16 h separation period. These data were not evaluated by statistical analysis, as they were casual observations rather than pre-designed experiments.

In studies on two separate groups of 6 rats (each comprising two cages of 3 rats), various perturbations were seen to impact on ambulatory activity and temperature (Fig 14). Following a routine (weekly) cage change, ambulatory activity recorded by the baseplates was increased for approximately 30–60 min. We also observed a brief increase in ambulatory activity coincident with an oral dosing procedure (Fig 14A). When rats were switched from group-housing to single-housing there was an immediate decrease in subcutaneous temperature which remained approximately 0.5°C lower throughout the 16 h period of single-housing, which spanned light and dark phases (Fig 14B).

## Discussion

### Optimal location for RFID transponder

Using both *ex vivo* and *in vivo* evaluations we have determined the optimum position–ventral abdominal midline–for an RFID transponder to allow continuous measurement of ambulatory activity and temperature in individual rats in group-housed situations. Validation of the automated readings for this implantation site found good concordance between baseplate-derived ambulatory activity and manual (birds-eye) tracking of ambulatory activity. It should be borne in mind that the automated tracking is truncated compared to the actual track of the animal, which will limit the degree of the concordance. This could also partly explain the drop-off in accuracy of tracking at higher levels of activity; the highest activity bin was the first 15 minutes following the cage change. It is conceivable that a combination of thigmotaxis (commonly seen when rats first enter and explore a ‘novel’ arena; [22]), the initial high speed of locomotion (which may be more difficult to track) and rearing (which elevates the height of the RFID transponder above the baseplate reader, and which is usually higher during initial exposure of rats to a new arena; [58]), will tend to impact on tracking accuracy.

Automated detection of vertical activity (defined by pixel movement above the horizontal cut-off line) was highly accurate when verified by manual assessment. However, vertical activity is not entirely equivalent to rearing, as evident by the presence of outliers when automated vertical activity was compared to manually verified rearing, but it does provide a strong correlate. Its current capability is equivalent to the conventional method of measuring ambulatory and vertical activity using two stacked arrays of photocell beams [15;17;18;19;20], but has the advantages of enabling group-housing in the home cage, 24/7 monitoring, with continuous measurement of subcutaneous temperature, and provision of 24/7 HD video for manual behavioral analysis.

### Sources of data variability

There was a small degree of variability in RFID read rate, recorded ambulatory activity and recorded temperature between different baseplates, but these were relatively insignificant in terms of biological significance, and are considered to be small in magnitude compared to the size of effects we would generally be interested in measuring. The system also revealed that baseline ambulatory activity differed between different cages of rats (quantified both via baseplate and video analytics). We have demonstrated the potential ability of the technology to detect effects on ambulatory activity and/or subcutaneous temperature caused by routine procedures commonly performed in rodent housing facilities (oral gavage dosing procedure; cage change; single-housing).

### Potential applications of the technology

As the effects of drugs can increase, diminish or remain the same on repeated dosing [27], various authors have encouraged the inclusion of behavioral endpoints in repeat-dose toxicology studies over the last three decades [27;59;60;61;62], and technology to facilitate this is long overdue. This new technology (ActualHCA™) has the potential to transform the way we do repeat-dose toxicity studies in rodents, and one likely deployment is in early repeat-dose toxicology studies in rats, where it would provide valuable additional data on adverse effects of new molecular entities on activity, behavior and temperature. Furthermore, in addition to toxicology and safety pharmacology, the technology has potential applications across the entire spectrum of behavioral neuroscience and drug discovery: behavioral phenotyping of transgenic animals [63;64]; CNS drug discovery [64;65], neurological and other disease models [31]; circadian biology [66]; drug dependence and withdrawal syndromes [67]; evaluation of environmental enrichment preference in rodent cages.

Work is ongoing to validate the current functionality pharmacologically, and to extend the behavioral recognition capability to both common (e.g., eating, drinking, grooming) and uncommon behaviors (e.g., convulsions), as has been achieved to varying extents in mice [68].

### 3Rs benefits

The ‘Rodent Big Brother’ project has delivered improvements to the quality and quantity of scientific data acquired from short- and long-term studies in rats, by combining the latest technological advances with 3Rs drivers [51;52;53;69]. Table 2 compares the functionality of Actual HCA™ with conventional technologies used in rats. The benefits include being able to greatly increase the information content and dimension of existing protocols by incorporating automated, continuous measurement of activity and temperature, and continuous capture of behavioral video, without disturbing the animals. The technology also has significant implications for animal welfare. The number of additional, standalone studies can be reduced, along with the number of animals used. Single housing of this social species can be avoided, minimising the potential anxiety caused. The ability to continuously monitor body temperature noninvasively also has significant advantages, removing the need for restraint or surgery and minimizing temperature fluctuations caused by stress.

**Table 2:** Anticipated advantages and drawbacks of Actual Home Cage Analyzer™ over existing technologies in rats

Parameter	Technology	Advantages	Drawbacks
Temperature	Rectal thermistor [42;47]	Relatively simple	Manual snapshot measurements only; requires manual restraint; not core
	Subcutaneous RFID transponder [42;48;49;50]	Relatively simple. Minimally invasive	Manual snapshot measurements only, using a hand-held RFID proximity reader; not core
	Infrared imaging of auditory canal [27]	Completely noninvasive. Approximates to core (at standard ambient housing temperatures)	Manual snapshot measurements only, using a thermal imaging camera. Will not work through wall of cage.
	Radiotelemetry [42]	24 h automated data acquisition; measures core temperature	Requires laparotomy surgery; expensive
	***Actual Home Cage Analyzer™***	***24 h automated measurements; animals undisturbed; minimally invasive***	***Subcutaneous temperature not core***
Ambulatory and vertical activity	‘Stabilimeter’ rocker cage [15;23]	Simple (switch activated as animal tips cage slightly moving around)	Requires single-housing; also requires soundproofing due to ‘clicking’
	Infrared photobeam arrays [15;16;17;18;19;20]	Reliable	Requires single-housing
	Videotracking [24;25;26;27]	Adaptable to different arenas; can track multiple animals independently using colour marking	Not feasible in standard home cages
	Vibration-sensitive platform [28;29;30]	Can detect a range of behaviours	Requires single-housing
	Passive infrared (PIR) sensor [21;22]	Relatively simple	Requires single-housing
	***Actual Home Cage Analyzer™***	***24 h automated measurements; enables individual tracking whilst group housed in the normal home cage*, *with bedding and environmental enrichment; conventional IVC housing on cage rack***	***Some loss of tracking at higher speeds of locomotion; vertical activity not entirely equivalent to rearing***

Importantly, the technology allows subtle changes in behavior, activity and temperature to be detected sooner and combined to improve the assessment of beneficial or adverse effects of compounds. More widely it could deliver global improvements in drug discovery and development through improved data quality, reduced and refined animal use, and increased efficiency.

## Supporting information

S1 Fig Understanding sources of variation in RFID transponder read rate from an *ex vivo* experiment. (DOCX)

S2 Fig Relationship between RFID transponder read rate and height above baseplate from the *ex vivo* experiment. (DOCX)

S3 Fig Understanding sources of variation in RFID transponder read rate *in vivo*. (DOCX)

S4 Fig Understanding sources of variation for ambulatory activity. (DOCX)

S5 Fig Understanding sources of variation for subcutaneous temperature. (DOCX)

S6 Fig Ambulatory activity validation for ventral midline RFID implantation site (pre-‘shielding upgrade’). (DOCX)

S7 Fig Ambulatory activity validation for flank: vertical RFID implantation site (pre-‘shielding upgrade’). (DOCX)

S8 Fig Ambulatory activity validation for flank: horizontal RFID implantation site (pre-‘shielding upgrade’). (DOCX)

S9 Fig Ambulatory activity validation for interscapular RFID implantation site (pre-‘shielding upgrade’). (DOCX)

S10 Fig Correlation plots of ambulatory movement of the rats derived from the baseplate RFID reader versus side-view pixel movement detection, for each of the 4 implantation sites. (DOCX)

S11 Fig Automated detection of individual rearing activity within a cage of 3 rats S1 Video Clip. Video clip illustrating view of cage containing 3 rats from side-view HD camera. The cage is illuminated by infrared lighting strips (visible at the top of the image) and contains a plastic play tunnel as part of the environmental enrichment; this is red in color but appears transparent in infrared lighting.
(DOCX)

S1 Video Clip 60 s video clip of 3 male Han Wistar rats co-housed in a Tecniplast SealSafe Blue-line individually ventilated cage (IVC). The infrared lighting strips are visible at the top of the image; the plastic play tunnel appears transparent in infrared light. The side-view, whole-cage video enables manual evaluation/quantification of behavior at any time of day or night. From the video, software quantifies whole-cage pixel movement as a measure of overall activity within the cage, and also detects vertical activity of individual rats.
(AVI)
