# Supplementary material for: Automated recording of home cage activity and temperature of individual rats housed in social groups: The Rodent Big Brother project
Source: PLoS One. 2017 Sep 6;12(9):e0181068. doi: 10.1371/journal.pone.0181068 (PMC5587114; doi:10.1371/journal.pone.0181068)
Supplement: S1 Fig — (DOCX) [file pone.0181068.s001.docx]

**Figure S1: Understanding sources of variation in RFID transponder read rate from an *ex vivo* experiment**Data were obtained using 2 RFID transponders across all 12 antennae for each of the 4 baseplates (designated ‘blue’, ‘green’, ‘red’ and ‘yellow’). The plot is relative to data from the ‘blue’ baseplate as reference. Each RFID transponder was positioned separately in a vertical orientation directly above center of an antenna, using a height-adjustable manipulator on top of the baseplate. Model diagnostics were explored and the model was found to be a good fit for the data (data not shown). This analysis reveals that the ‘shielding upgrade’ increased the read rate on average by 0.07 Hz; for each 1 cm decrease in height the read rate decreases by 0.08Hz, and the ‘red’ baseplate gave readings that were on average -0.05 Hz lower. The open circles are the mean values, the bars are the 95% confidence intervals.
