# Supplementary material for: Automated recording of home cage activity and temperature of individual rats housed in social groups: The Rodent Big Brother project
Source: PLoS One. 2017 Sep 6;12(9):e0181068. doi: 10.1371/journal.pone.0181068 (PMC5587114; doi:10.1371/journal.pone.0181068)
Supplement: S2 Fig — (DOCX) [file pone.0181068.s002.docx]

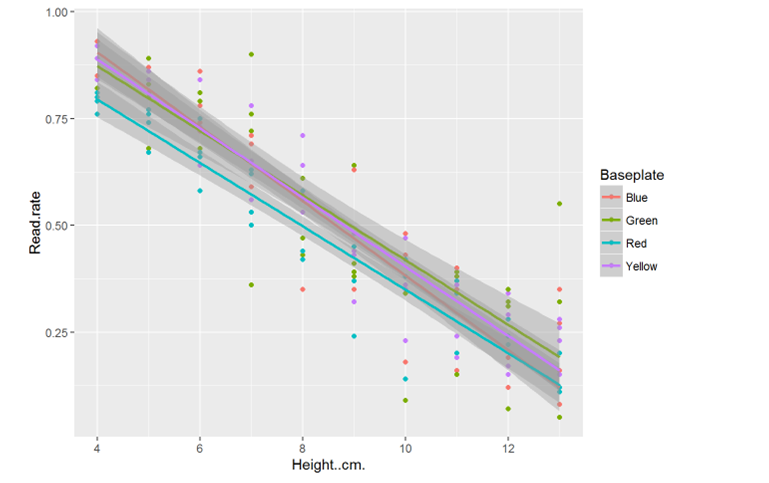


**Figure S2: Relationship between RFID transponder read rate and height above baseplate from the ex vivo experiment**

The regression analysis assumes a linear relationship between height and read rate. A linear relationship was obtained for all 4 baseplates (designated ‘blue’, ‘green’, ‘red’ and ‘yellow’). The gray region is the 95% confidence interval for the fitted line.
