# Supplementary material for: Automated recording of home cage activity and temperature of individual rats housed in social groups: The Rodent Big Brother project
Source: PLoS One. 2017 Sep 6;12(9):e0181068. doi: 10.1371/journal.pone.0181068 (PMC5587114; doi:10.1371/journal.pone.0181068)
Supplement: S3 Fig — (DOCX) [file pone.0181068.s003.docx]

**Figure S3: Understanding sources of variation in RFID transponder read rate *in vivo***

Data were obtained by rotating two cages of 3 rats for each implantation site across each of the 4 baseplates, for 7 consecutive days on each baseplate. Model diagnostics were explored and the model was found to be a good fit for the data (data not shown). The ‘shielding upgrade’ significantly increased read rate (by 0.14 ±0.21 Hz). The implantation site was a significant source of variation. When the ventral midline site is set as the reference, all 3 other locations had a decrease in read rate relative to the midline. For example, the read rate from the flank: horizontal position decreased by 0.30 ± 0.04 Hz compared to the ventral midline position. Again, read rate varied between baseplates, with ‘red’ leading to a decrease (-0.053 ± 0.013 Hz) and green having a small increase (0.034 ± 0.012 Hz) compared to the ‘blue’ baseplate as reference. The open circles are the mean values, the bars are the 95% confidence intervals.
