# Supplementary material for: Automated recording of home cage activity and temperature of individual rats housed in social groups: The Rodent Big Brother project
Source: PLoS One. 2017 Sep 6;12(9):e0181068. doi: 10.1371/journal.pone.0181068 (PMC5587114; doi:10.1371/journal.pone.0181068)
Supplement: S4 Fig — (DOCX) [file pone.0181068.s004.docx]

**Figure S4: Understanding sources of variation for ambulatory activity**

Analyses on sources of variation of ambulatory activity (transitions) data when data summed in 15-minute bins *in vivo* using the ventral midline implantation site, following the ‘shielding upgrade’. Ambulatory activity is computed from the transitions read from the baseplate. Data were obtained by rotating two cages of 3 rats across each of the 4 baseplates, for 7 consecutive days over each baseplate (D = Day; W = Week). The plot is relative to data from Day 1, Week 1 and ‘blue’ baseplate, respectively, as reference. ‘Phase’ indicates the decrease in activity in the light phase compared to the dark phase. Model diagnostics were explored and the model was found to be a good fit for the data (data not shown). The number of transitions within a 15 minute bin varied by less than 5 for day or week of study. Relative to the ‘blue’ baseplate, the other 3 baseplates varied by less than 5 transitions within a 15 minute bin. This evaluation indicates that the technology is capable of detecting changes of this magnitude (> ~5 transitions per 15 minutes). The open circles are the mean values, the bars are the 95% confidence intervals.
