# Supplementary material for: Automated recording of home cage activity and temperature of individual rats housed in social groups: The Rodent Big Brother project
Source: PLoS One. 2017 Sep 6;12(9):e0181068. doi: 10.1371/journal.pone.0181068 (PMC5587114; doi:10.1371/journal.pone.0181068)
Supplement: S5 Fig — (DOCX) [file pone.0181068.s005.docx]

**Figure S5: Understanding sources of variation for subcutaneous temperature**

Regression analysis to understand sources of variation of *in vivo* temperature data when averaged across 15-minute bins using the ventral midline implantation site, following the ‘shielding upgrade’. Data were obtained by rotating two cages of 3 rats across each of the 4 baseplates, for 7 consecutive days over each baseplate. Model diagnostics were explored and the model was found to be a good fit for the data (data not shown). The analysis found: temperatures were 0.25°C lower during the light phase; Day 1 has a higher temperature by around 0.05°C compared to other days; Week 1 by around 0.1°C compared to other weeks, and the ‘red’ and ‘yellow’ baseplates  gave 0.05°C and 0.08°C lower readings, respectively. As the RFID transponders are factory precalibrated, their temperature data is read digitally by the baseplates, and the data in the above analysis is from 4 cages of rats rotated across the baseplates, these small differences in temperature are possibly due to differences in ambient temperature within the IVC cages at different positions on the IVC rack. Nonetheless, this evaluation indicates that the technology is capable of detecting temperature changes of this magnitude (>~0.1°C). The open circles are the mean values, the bars are the 95% confidence intervals.
