# Supplementary material for: Automated recording of home cage activity and temperature of individual rats housed in social groups: The Rodent Big Brother project
Source: PLoS One. 2017 Sep 6;12(9):e0181068. doi: 10.1371/journal.pone.0181068 (PMC5587114; doi:10.1371/journal.pone.0181068)
Supplement: S7 Fig — (DOCX) [file pone.0181068.s007.docx]

a


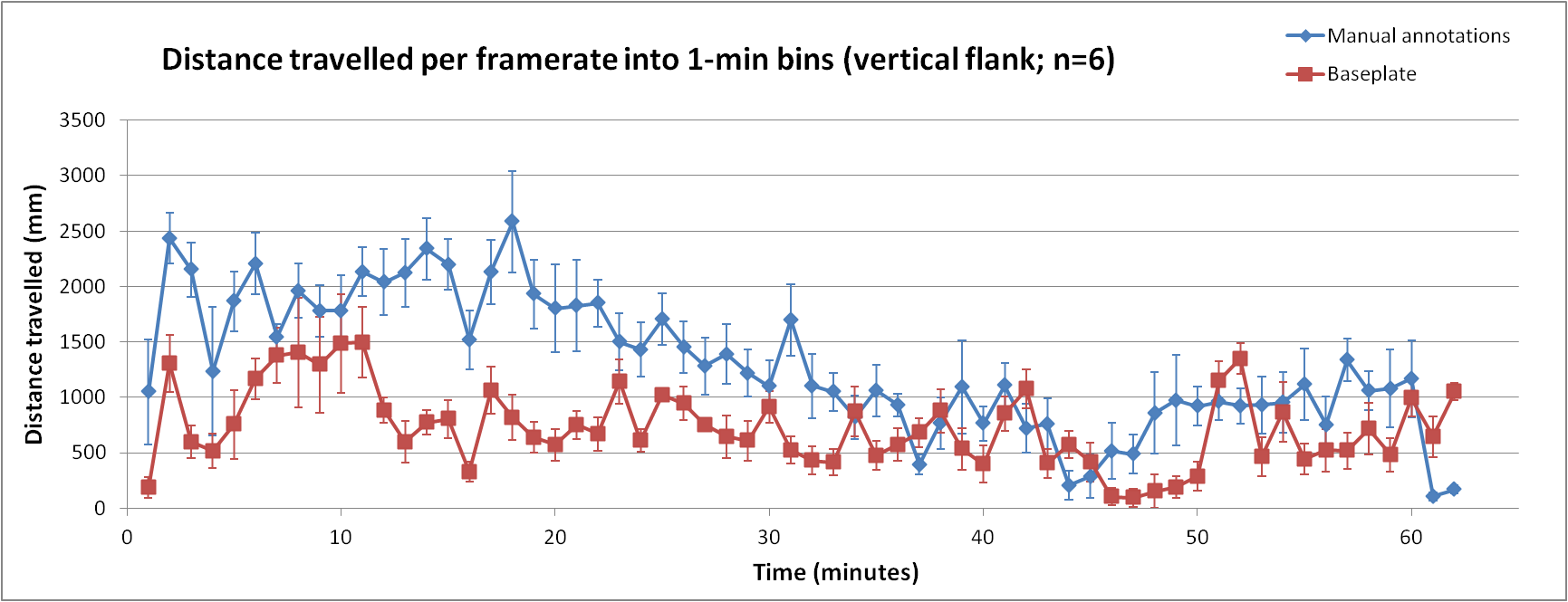


b c

**Figure S7:** **Ambulatory activity validation for flank: vertical RFID implantation site (pre-‘shielding upgrade’)**

(a) Ambulatory movement of the rats derived from the baseplate RFID reader (red line) compared with movement assessed by manual tracking (blue line) over a 60 min period (plotted in 1-minute bins; mean of 6 rats ± SEM), for the flank: vertical implantation site. (b) Correlation between baseplate and manual activity data (distance travelled) from the 6 rats plotted in 15-minute bins. The dotted line is the line of unity. ICC = -0.01. (c) Bland-Altman plot showing the average difference between the baseplate and manual measurements. There is sub-optimal concordance between automated and manual tracking. This concordance was improved (to an ICC of 0.61) following the ‘shielding upgrade’ (data not shown).
