# Supplementary material for: Automated recording of home cage activity and temperature of individual rats housed in social groups: The Rodent Big Brother project
Source: PLoS One. 2017 Sep 6;12(9):e0181068. doi: 10.1371/journal.pone.0181068 (PMC5587114; doi:10.1371/journal.pone.0181068)
Supplement: S10 Fig — (DOCX) [file pone.0181068.s010.docx]

**Figure S10:** **Correlation plots of ambulatory movement of the rats derived from the baseplate RFID reader versus side-view pixel movement detection, for each of the 4 implantation sites**

Each point is a 30-minute average for the mean of 3 rats plotted against whole cage pixel movement over a 3-4 week period (2 cages of 3 rats per plot; total of 2150 to 2686 data points on each plot). (a-d) Data obtained prior to the ‘shielding upgrade’: (a) flank: vertical; (b) ventral midline; (c) flank: horizontal; (d) interscapular. The best two performing sites were re-tested post-‘shielding upgrade’: (e) flank: vertical and (f) ventral midline.
