# Supplementary material for: Automated recording of home cage activity and temperature of individual rats housed in social groups: The Rodent Big Brother project
Source: PLoS One. 2017 Sep 6;12(9):e0181068. doi: 10.1371/journal.pone.0181068 (PMC5587114; doi:10.1371/journal.pone.0181068)
Supplement: S11 Fig — Video clip illustrating view of cage containing 3 rats from side-view HD camera. The cage is illuminated by infrared lighting strips (visible at the top of the image) and contains a plastic play tunnel as part of the environmental enrichment; this is red in color but appears transparent in infrared lighting. (DOCX) [file pone.0181068.s011.docx]

**Figure S11:** **Automated detection of individual rearing activity within a cage of 3 rats**

Upper panel: Two broken column charts showing the automated detection of vertical activity of a 15-min bin each from the light and dark phase video footage. Red column indicates the time spent in vertical activity for the whole cage. Green, purple and cyan column charts show each individual rat in the cage. Bar chart shows the number of rearing episodes by manual counting for each animal in the light and dark phases.
